# Surgical Management of a Pancreaticopleural Fistula After Failed Endoscopic Therapy

**DOI:** 10.7759/cureus.23241

**Published:** 2022-03-17

**Authors:** Nikolaos Koliakos, Dimitrios Papakonstantinou, Lazaros Reppas, Anargyros Bakopoulos, Andrianos Tzortzis, Dimitrios Polymeros, Nikolaos Oikonomopoulos, Emmanouil Pikoulis, Georgios Martikos

**Affiliations:** 1 Third Department of Surgery, Attikon University General Hospital/National and Kapodistrian University of Athens, School of Medicine, Athens, GRC; 2 Interventional Radiology Unit, Second Department of Radiology, Attikon University General Hospital/National and Kapodistrian University of Athens, School of Medicine, Athens, GRC; 3 Hepatogastroenterology Unit, Second Department of Pathology, Attikon University General Hospital/National and Kapodistrian University of Athens, School of Medicine, Athens, GRC; 4 Second Department of Radiology, Attikon University General Hospital/National and Kapodistrian University of Athens, School of Medicine, Athens, GRC

**Keywords:** case report, endoscopic therapy, thoracopancreatic fistula, pancreaticopleural fistula, surgical management

## Abstract

Inflammatory diseases of the pancreas or pancreatic trauma result in ductal cell disruption, which in turn may lead to leakage of pancreatic fluid, mostly in the retroperitoneal space. Pancreatopleural fistulas are uncommonly encountered following pancreatic injury; however, they often prove a difficult problem to manage.

Herein, we present a rare case of a 68-year-old male suffering from a pancreaticopleural fistula (PF) between the pancreatic tail and the left pleural space one year following splenectomy for trauma. About three months after percutaneous drainage of a left pleural effusion and left upper quadrant abdominal collection and endoscopic pancreatic duct stent placement, surgical management was decided. Distal pancreatectomy and Roux-en-Y drainage of the pancreatic remnant were successfully performed.

## Introduction

Pancreatic inflammatory diseases causing acinar and ductal cell dysfunction sometimes result in disastrous complications [1]. Pancreaticopleural fistula (PF) is an extremely rare complication of both acute and chronic pancreatitis as well as pancreatic trauma with duct disruption [1,2]. Pancreatic fluid may enter the pleural cavity either directly through diaphragmatic pores or through the aortic and/or esophageal hiatus, leading to a massive exudative pleural effusion [2].

## Case presentation

A 68-year-old Caucasian male patient presented to the emergency department with shortness of breath. His past medical history included smoking and hypertension. His past surgical history was significant for traumatic splenectomy one year before the beginning of his complaints. On chest radiography, a massive left pleural effusion was demonstrated, and the patient underwent thoracocentesis, which drained 1 L of milky pleural fluid. Biochemical analysis of the pleural fluid revealed amylase of 7,432 IU/dL, which raised concerns for the presence of a pancreatic ductal communication with the left pleural cavity. An injury in the pancreatic tail during splenectomy was suspected as the cause of the thoracopancreatic fistula.

**Figure 1:**
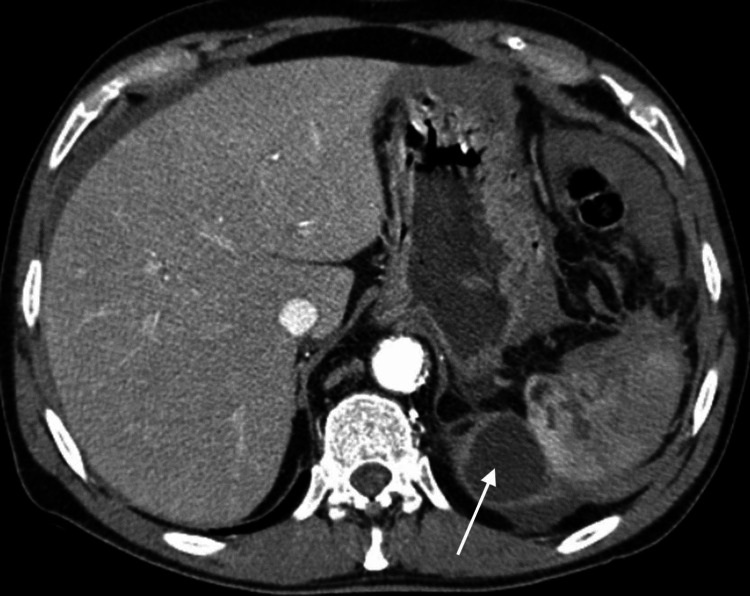
CT scan demonstrating an abdominal pancreatic collection at the anatomic site of the removed spleen (white arrow)

Two days after his admission, the patient presented hemoptysis. Computed tomography revealed a 5-cm thin-walled cyst in the pancreatic tail and persistent pleural effusion (Figure 1). Endoscopic retrograde cholangiopancreatography (ERCP) was thereafter performed, demonstrating contrast extravasation from the main pancreatic duct stump. Following sphincterotomy, a 5 Fr plastic stent was placed, and thereafter, a drainage catheter was percutaneously inserted near the fluid collection at the pancreatic tail. Following these procedures, the pleural effusion was entirely drained with no evident persistent collection after the removal of the pleural cavity drain. On his first follow-up appointment three months after the ERCP, the patient noted persistent drainage of 100 (or more) mL of turbid fluid per day from his abdominal drain.

**Figure 2:**
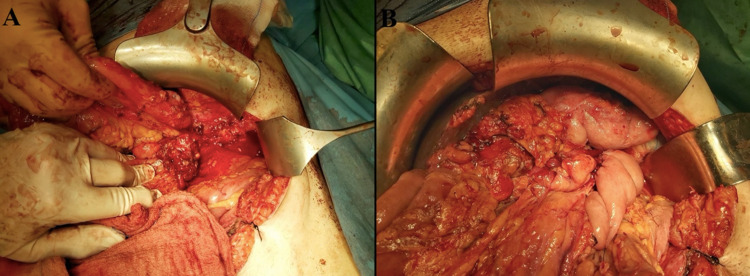
Intraoperative pictures after distal pancreatectomy (A) and following Roux-en-Y reconstruction (B)

A new contrast-enhanced computer tomography demonstrated the formation of pancreatic stones inside the main pancreatic duct and, simultaneously, an inflammatory process in the pancreatic body and tail fistulizing in the left pleural space. Due to the failure of the conservative management, a laparotomy was decided. The peritoneal cavity was entered through a midline incision, and adhesions between the pancreas and the surrounding structures were taken down. The fistula between the pancreatic tail and the left hemidiaphragm could not be visualized because of the advanced inflammatory process. Distal pancreatectomy and Roux-en-Y pancreaticojejunostomy with invagination of the pancreatic remnant were performed (Figure 2). The operation time was 130 minutes, and the blood loss during surgery was 150 mL.

His postoperative course was uneventful. He was discharged on the 14th postoperative day. The histopathological examination of the pancreatic parenchyma showed irregular fibrosis and inflammatory cell infiltrates compatible with chronic pancreatitis. He remains in good health 12 months after the procedure.

## Discussion

Pancreatitis fluid collections, caused by disruptions of the pancreatic duct, may cross over into the pleural space via the diaphragmatic hiatuses or via subperitoneal lymphatics situated on the left hemidiaphragm. Dyspnea, cough, and chest pain are the most frequent presenting symptoms in such cases [2]. Pathologic accumulation of fluid within the pleural space can be appreciated in imaging studies, and high concentrations of fluid amylase level can help steer the physician toward the diagnosis of a PF.

The diagnosis and management of PF remain individualized depending on patient status, pancreatic pathology, and fistula anatomy. In this setting, the role of endoscopic retrograde cholangiopancreatography (ERCP) as a diagnostic and therapeutic modality is increasingly expanded. Hence, surgical management is most useful as a salvage procedure [2].

A significant step in pancreatic fistula management is to precisely define pancreatic ductal anatomy. Noninvasive magnetic resonance cholangiopancreatography (MRCP) is considered a valuable imaging method resulting in an accurate diagnosis in up to 83% of cases [3]. Nevertheless, it is essential to emphasize the diagnostic and curative potential of endoscopic retrograde cholangiopancreatography (ERCP) regarding pancreatic ductal leaks and fistulas [2]. In our case, sphincterotomy and pancreatic stent placement eventually led to pancreatic head ductal stone formation, thus increasing pressures in the terminal part of the pancreatic duct, consequently exacerbating drainage outflow volumes from the pancreatic tail and into the thorax.

Surgical treatment is recommended when conservative treatment methods fail to improve the patient’s condition and eliminate pancreatic leakage [2]. Distal pancreatectomy, Roux-en-Y pancreatic duct drainage, and cystojejunostomy have been proposed depending on the location of pancreatic ductal disruption [4]. Due to the presence of multiple pancreatic duct calculi, we decided to combine distal pancreatectomy and end-to-side Roux-en-Y pancreatic remnant drainage. To the best of our knowledge, four cases of pancreaticopleural fistula have been treated similarly [5-8], while Heiss et al. decided to anastomose the divided pancreatic duct to the posterior gastric wall [9]. No recurrence of pleural effusion was recorded among those cases, whereas one of them developed a grade A postoperative fistula [5].

## Conclusions

Pancreatopleural fistula is a rare complication of pancreatic disease associated with inflammation, trauma, or iatrogenic injury. The patient’s history and comprehensive clinical examination are indispensable for the differential diagnosis of recurrent pleural effusions. A high index of suspicion should be maintained, especially when brownish thick amylase-rich fluid is drained. A multidisciplinary approach is recommended in every case, using a step-up approach from conservative to endoscopic management, with surgery reserved as salvage in intractable cases.
